# Hypertension Programmed in Adult Hens by Isolated Effects of Developmental Hypoxia In Ovo

**DOI:** 10.1161/HYPERTENSIONAHA.120.15045

**Published:** 2020-06-15

**Authors:** Katie L. Skeffington, Christian Beck, Nozomi Itani, Youguo Niu, Caroline J. Shaw, Dino A. Giussani

**Affiliations:** 1 From the Department of Physiology, Development and Neuroscience, University of Cambridge, United Kingdom (K.L.S., C.B., N.I., Y.N., C.J.S., D.A.G.); 2 Department of Metabolism, Digestion and Reproduction, Institute of Reproductive and Developmental Biology, Imperial College London, United Kingdom (C.J.S.).

**Keywords:** baroreflex, cardiovascular diseases, hypertrophy, hypoxia, pregnancy

## Abstract

In mammals, pregnancy complicated by chronic hypoxia can program hypertension in the adult offspring. However, mechanisms remain uncertain because the partial contributions of the challenge on the placenta, mother, and fetus are difficult to disentangle. Here, we used chronic hypoxia in the chicken embryo—an established model system that permits isolation of the direct effects of developmental hypoxia on the cardiovascular system of the offspring, independent of additional effects on the mother or the placenta. Fertilized chicken eggs were exposed to normoxia (N; 21% O_2_) or hypoxia (H; 13.5%–14% O_2_) from the start of incubation (day 0) until day 19 (hatching, ≈day 21). Following hatching, all birds were maintained under normoxic conditions until ≈6 months of adulthood. Hypoxic incubation increased hematocrit (+27%) in the chicken embryo and induced asymmetrical growth restriction (body weight, −8.6%; biparietal diameter/body weight ratio, +7.5%) in the hatchlings (all *P*<0.05). At adulthood (181±4 days), chickens from hypoxic incubations remained smaller (body weight, −7.5%) and showed reduced basal and stimulated in vivo NO bioavailability (pressor response to NG-nitro-L-arginine methyl ester, −43%; phenylephrine pressor response during NO blockade, −61%) with significant hypertension (mean arterial blood pressure, +18%), increased cardiac work (ejection fraction, +12%; fractional shortening, +25%; enhanced baroreflex gain, +456%), and left ventricular wall thickening (left ventricular wall volume, +36%; all *P*<0.05). Therefore, we show that chronic hypoxia can act directly on a developing embryo to program hypertension, cardiovascular dysfunction, and cardiac wall remodeling in adulthood in the absence of any maternal or placental effects.

## Abstract 2

Supplemental Digital Content is available in the text.

Hypertension is a risk factor for adverse cardiovascular events.^1,2^ It is well known that an individual’s risk of developing hypertension is affected by genetics, lifestyle choices, and the interaction between the two.^3,4^ However, it is now accepted that hypertension in adulthood can also be induced by exposure to adverse environmental conditions before birth, which alter fetal development in a way that predisposes the individual in later life to a higher risk of cardiovascular dysfunction, what is known as developmental programming.^5,6^ Examples of adverse environmental conditions in utero, which can program hypertension, include nutritional deficits,^7,8^ glucocorticoid treatment,^9^ and exposure to toxins such as tobacco.^10^ However, in this study, we focus on chronic fetal hypoxia, as it is one of the most common outcomes of complicated pregnancy in humans.^11–15^

Studies using mammalian animal models, mostly in rodents and sheep, have shown that hypoxic pregnancy can program hypertension in the adult offspring via alterations in NO biology and sympathetic reactivity in the cardiovascular system.^16–18^ Such studies, together with ours, have used several techniques to induce chronic fetal hypoxia, including hypoxic chambers,^17,19–21^ uterine artery blood flow restriction,^16,22^ placental embolization,^23,24^ umbilical artery ligation,^25^ carunclectomy,^26^ and exposure to high altitude.^27–29^ Methods such as uterine artery restriction, placental embolization, or umbilical artery ligation will also reduce nutrient transfer to the fetus, and pregnant mammals placed in hypoxic chambers sometimes show reduced food intake,^30–32^ although this is not always the case.^20,33^ Some studies have attempted to control for hypoxia-induced reductions in maternal food intake by using a separate group of pair-fed animals.^31,32,34^ However, this only gives an indication of the combined effects of undernutrition and hypoxia, compared with undernutrition alone. The isolated programming effects of developmental hypoxia are harder to elucidate. Another limitation of mammalian models is that developmental programming of the offspring can occur secondary to effects of the adverse environment on the placenta and the mother. This complicates the isolation of underlying mechanisms and thereby identification of plausible therapeutic targets for intervention.

In oviparous species, such as in chickens, embryonic development can occur in the absence of either a mother or a placenta, and all available nutrition is fixed within the egg from the start of incubation. There is no need to consider within-litter variation or effects on lactation. Added value is that the temporal milestones of cardiovascular development in humans are more similar to the chicken than to rodents, the latter being born highly immature and cardiovascular development continuing for weeks past birth.^35^ Avian species are, therefore, ideally placed to isolate the direct programming effects of developmental hypoxia on the offspring’s cardiovascular system and underlying genes and signaling pathways. Therefore, using an integrative approach by combining invasive and noninvasive experiments in vivo with those in isolated organs and at the cellular level, in this study, we have tested the hypothesis that developmental hypoxia alone can directly program hypertension in the adult offspring. Further, mechanisms underlying this effect include impaired NO biology and enhanced sympathetic cardiac reactivity.

## Methods

### Data, Materials, and Code Disclosure Statement

The data that support the findings of this study are available from the corresponding author upon reasonable request.

### Ethical Approval

This research was approved under the Animals (Scientific Procedures) Act 1986 Amendment Regulations 2012 following ethical review by the University of Cambridge Animal Welfare and Ethical Review Board.

### Animals

Fertilized Bovans Brown chicken eggs (Gallus gallus domesticus) were purchased from Medeggs (Henry Stewart & Co, Norfolk, United Kingdom). This supplier delivers fertilized eggs in batches from a single day’s laying, meaning any one egg used in the present programme of work came from a different hen. Fertilized eggs were weighed and incubated under normoxic (21% O_2_) or hypoxic (13.5%–14% O_2_) conditions (37.9°C, 45% humidity, 12:12-hour light:dark cycle, automatic rotation every hour; incubator Models 75-A and M-240, with humidifier THP 1100-03239 and Humidity kit HS Auto-3.5 L; all Masalles, Barcelona, Spain). The levels of oxygen, temperature, and humidity inside the incubators were all continuously monitored (DD103 DrDAQ Oxygen Sensor; Pico Technology, St Neots, United Kingdom). Eggs were candled on days 13 and 19 (hatching occurs at ≈day 21) to assess viability. On day 19, one cohort of embryos was used to determine measurements in blood. Values for hematocrit were obtained using a microhematocrit centrifuge, in duplicate (Hawksley, United Kingdom). Blood glucose and lactate concentrations were measured using a glucose/lactate analyzer (Yellow Springs 2300 Stat Plus Glucose/Lactate Analyzer; YSI, Ltd, Farnborough, United Kingdom). The remaining embryos were allowed to hatch in a normoxic (21% O_2_) incubator (37.9°C, 60% humidity, 12:12-hour light:dark cycle). The hatchlings were weighed, crown rump length (the distance from the external occipital protuberance to the base of the tail) was measured using a measuring tape and biparietal diameter (BPD; the distance between the outside corner of each eye), and the lengths of each leg bone were measured using digital calipers. The chicks were placed in a brooder (35°C) for 2 to 3 weeks before being moved into a barn (room temperature). Chickens that had been exposed to normoxic or hypoxic incubation lived in mixed groups and had ad libitum access to food and water. Regular measurements of body weight and crown rump length were recorded as they grew to adulthood, which we defined as 6 to 7 months of age, shortly after the age at which hens start laying eggs.^36^ Body mass index was calculated from these measurements. To control for sex differences, only female chickens were kept until adulthood and studied. Tissues from male chickens were isolated at 5 weeks for future experiments.

### In Vivo Experiments

#### Echocardiography

Conscious adult chickens were gently held by an investigator and restrained in the supine position. A second investigator then used a Toshiba PowerVision 7000 ultrasound machine with a phased array 2.0 MHz probe (PSK-20CT) to determine the function of the left ventricle (LV), right ventricle, and valves. Ejection fraction and fractional shortening were calculated using the Teichholz method.^37^

#### Chronic Instrumentation

A subset of chickens was anesthetized (2%–2.5% Isoflurane in 4 L O_2_/min [IsoFlo; Abbott Laboratories, Ltd, Berkshire, United Kingdom]) and surgically instrumented with 1 femoral arterial catheter, 2 femoral venous catheters, and a femoral arterial Transonic flow probe on the contralateral leg, as described in detail elsewhere.^38^ Following 5 days of recovery from surgery, basal blood gases, pH and blood glucose, and lactate values were taken (ABL5 and ABL80 from Radiometer, Copenhagen, Denmark, and a glucose/lactate analyzer from Yellow Springs 2300 Stat Plus, YSI, Ltd, Farnborough, United Kingdom). Femoral oxygen and glucose delivery (content×flow) were calculated.^39^ To determine in vivo cardiovascular function, chickens were placed into a sling within a dark wooden box with air vents. With their feet off the floor, the chickens tended to fall asleep, permitting recording of in vivo basal and stimulated cardiovascular function. Data were recorded via connection of the arterial catheter to a saline-filled pressure transducer (Argon Medical Devices, Plano, TX), which was in turn connected to an M-PAQ system (Maastricht-Programmable AcQuisition System [1000 Hz sample rate] with IDEEQ software; Maastricht Instrument, Maastricht, the Netherlands). The flow probe was connected to a Transonic meter (T206; Transonic Systems, Ithaca, NY). Four experiments were performed on different days, all reagents were obtained from Sigma-Aldrich (Gillingham, United Kingdom) unless stated otherwise, and all drugs made up in sterile saline. Experiments consisted of (1) intravenous administration of the α_1_-adrenergic receptor agonist phenylephrine (PE) in bolus doses (16, 32, 64, 96, and 128 µg·kg^−1^) in a randomized order; (2) intravenous administration of the vascular smooth muscle–dependent dilator sodium nitroprusside (SNP) in bolus doses (1, 5, 10, 15, and 20 µg·kg^−1^) in a randomized order; (3) a single intravenous dose of the eNOS (endothelial NO synthase) inhibitor NG-nitro-L-arginine methyl ester (L-NAME; 100 mg·kg^−1^; Cayman Chemicals, Cambridge, United Kingdom); and (4) exposure to an NO clamp, followed by PE treatment. While administration of a single dose of L-NAME permits the contribution of basal NO bioavailability to be determined, the NO clamp is a technique validated in vivo,^40–42^ which permits the contribution of stimulated de novo synthesis of NO bioavailability to be determined without perturbation to basal cardiovascular function. To achieve the NO clamp, an intravenous SNP infusion was started (2 µg·kg^−1^·min^−1^; infusion volume, 0.15 mL·min^−1^), and a bolus dose of L-NAME (100 mg·kg^−^^1^) was given via the second venous catheter just before the SNP reached the chicken circulation. The rate of SNP infusion was titrated to maintain mean arterial blood pressure (MAP) and heart rate at basal values. Once clamped and under conditions of NO blockade while maintaining basal cardiovascular function, a single dose of PE (64 µg·kg^−1^) was then administered via the second intravenous catheter. At the end of the experiment, the SNP infusion was stopped, and recording continued for ≈10 minutes to ensure that arterial blood pressure increased, thereby validating the effectiveness of the clamp in blocking NO synthesis and the persistence of bioactive L-NAME in the system.^40–42^

#### Baroreflex Function

Cardiovascular data were transferred into Labchart (Labchart Pro, version 7.3.1; AD Instruments, Chalgrove, United Kingdom) for analysis. Basal cardiovascular function was determined, and the cardiovascular responses to drugs calculated by comparing the maximal blood pressure and heart rate change from stable baseline just before administration of each agonist. To generate baroreflex curves, the heart rate and blood pressure responses to the SNP and PE doses were plotted against each other and a 4-parameter logistic function applied.^43^ Baroreflex gain was then calculated for the average baroreflex curve for each treatment group using the following equation: Gain=Hill slope×(HR_max_−HR_min_)/4.^43^

### Ex Vivo Experiments

Chickens were killed by intravenous administration of 2 mL sodium barbiturate. There was no significant difference between the 2 groups for the age at which the chickens were killed (N, 178±5 days; H, 186±5 days; *P*>0.05). Crown rump length, BPD, and leg lengths were measured as described above, and organs were dissected and weighed. One cohort was reserved for cardiac stereology and a second cohort for determining cardiac function in a Langendorff preparation.

#### Cardiac Stereology

One cohort of chickens underwent perfusion fixation. The heart was exposed, a cut made in the right atrium and heparinized saline (100 IU heparin per mL saline, heparin sodium; Wockhardt, Wrexham, United Kingdom) passed into the LV through a needle from a height of 170 cm. The chicken was then perfused with a solution of paraformaldehyde (Formal Fixx 10% Neutral Buffered Formalin; Thermo Fisher Scientific, Loughborough, United Kingdom) at a physiological pressure of 16.8 kPa, after which the heart was harvested and stored in 10% paraformaldehyde for 3 days before transfer to PBS. Perfusion fixed hearts were cut into slices 3-mm thick. Photographs were taken and analysis performed using ImageJ (version 1.46; National Institutes of Health, Bethesda, MD). Values for the left and right ventricular wall and lumen areas of a midcardiac cross section were determined by superimposing a point grid onto the sections, and wall width was measured. Left and right ventricular wall and lumen volumes of the whole heart were estimated using Cavalieri Principle.^44^

#### Langendorff Preparation

A second cohort of chickens was administered 5000 IU intravenous heparin (5000 IU) in saline immediately before death. Hearts were dissected, mounted on a Langendorff apparatus, and perfused with Krebs solution (in mmol·L^−1^: NaCl 118.5, NaHCO_3_ 25, KCl 4.7, MgSO_4_·7H_2_O 1.2, KH_2_PO_4_ 1.2, CaCl_2_ 1.9, D-glucose 13.9), which was filtered (glass Fiber Prefilter and 8 μm membrane filter; both from Millipore, Watford, United Kingdom), heated to 38°C, and aerated with 95% O_2_/5% CO_2_. Pressure generated by the heart was measured via insertion of a saline-filled, nonelastic balloon into the LV via a small incision in the left atrium. The balloon was connected via a rigid saline-filled catheter to a pressure transducer (Argon Medical Devices, Plano, TX), which was in turn connected to an M-PAQ system (Maastricht-Programmable AcQuisition System [1000 Hz sample rate] with IDEEQ software; Maastricht Instrument). Following stable baseline, doses of the _1_-adrenergic receptor agonist isoprenaline (1×10^−9^ to 1×10^−7^ mol·L^−1^) and the muscarinic receptor agonist carbamylcholine chloride (1×10^−8^ to 1×10^−6^ mol·L^−^^1^) were administered. The data were exported to Labchart for analysis of basal cardiac function using basic Labchart functions and the blood pressure analysis module. The maximal heart rate responses to the agonist doses were then calculated as a percentage of the baseline immediately before each dose.

#### Wire Myography

From the same animals used for the Langendorff preparation, second-order femoral arteries were dissected and mounted in Krebs solution (in mmol·L^−1^: NaCl 118.5, NaHCO_3_ 25, KCl 4.7, MgSO_4_·7H_2_O 1.2, KH_2_PO_4_ 1.2, CaCl_2_ 2.5, D-glucose 2.8, 37°C, 95% O_2_) on a 4-chamber wire myograph (610M; Danish Myo Technology, Aarhus, Denmark). Vessels were normalized to 90% of 16.8 kPa. The responses to 6 doses of potassium (in mmol·L^−^^1^: 16.74, 28.77, 40.80, 64.86, 125, and 250), PE (1×10^−9^ to 1×10^−4^ M half log increments), SNP (1×10^−10^ to 1×10^−4^ mol·L^−^^1^, whole log increments), and acetylcholine (1×10^−9^ to 1×10^−4^ mol·L^−^^1^, half log increments) were investigated, leaving at least 20 minutes between curves. The SNP and acetylcholine curves were performed following preconstriction with a suboptimal dose of K^+^ (response <85% of the maximal K^+^ response). The acetylcholine curve was repeated in the presence of L-NAME (1×10^−5^ mol·L^−1^; Cayman Chemical, Cambridge, United Kingdom) to determine the contribution of NO-dependent and NO-independent mechanisms to acetylcholine-mediated vasorelaxation.^45^

### Data and Statistical Analysis

All experiments were performed for normoxic and hypoxic groups in parallel, and investigators for any one outcome were blinded to treatments, which were coded. Femoral vascular resistance and femoral vascular conductance were calculated following the Ohm principle. Vascular resistance was calculated by dividing arterial pressure by flow. Vascular conductance was calculated by dividing flow by arterial pressure. All values are presented as mean±SEM. Data were tested for normality using the Kolmogorov-Smirnov test, and transformations applied if necessary. Comparisons between 2 groups were made with the 2-tailed Student *t* test for unpaired data (SigmaStat, version 3.5; Systat Software, San Jose, CA). In one case where the data could not be normalized, the Mann-Whitney *U* test was used. The Student *t* test with Welch correction was applied to data, which were normally distributed but had unequal variances. Survival data were analyzed with the χ^2^ test (SPSS, version 23; IBM Analytics, New York, NY). For experiments with a repeated measures factor (time or dose), a 2-way mixed-model ANOVA test was performed in SPSS. For any mixed-model ANOVA tests, the false discovery rate was controlled for,^46^ and sphericity was tested using Maunchly test. Where the assumption of sphericity was violated, the appropriate correction was made to the *df*. Basal arterial blood pressure at adulthood was related to biometry at hatching. Correlations were tested using the 2-tailed Pearson correlation coefficient (GraphPad Prism, version 5.0; Graphpad Software, Inc, San Diego, CA). For all statistical comparisons, significance was accepted when *P* was <0.05.

## Results

### Survival, Blood Chemistry, and Growth

**Figure 1.**
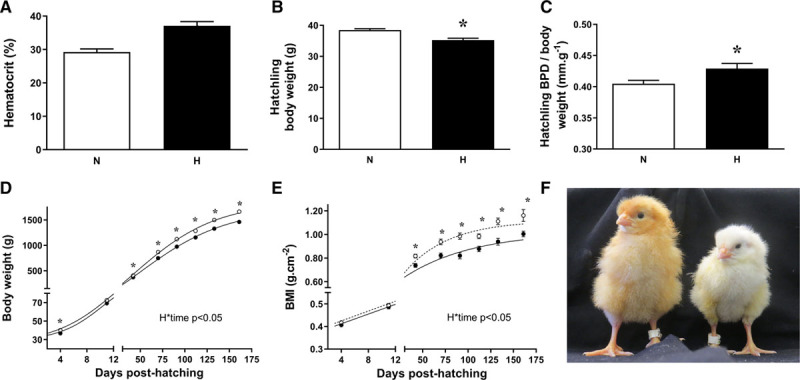
Hematocrit and biometry. Values are mean±SEM for the chicken embryo hematocrit at day 19 incubation (**A**), the body weight at hatching (**B**), the biparietal diameter (BPD)/body weight ratio at hatching (**C**), the body weight post-hatching growth curve (**D**), the body mass index (BMI) post-hatching growth (**E**) and a photo of representative chicks at hatching (**F**; N, left; H, right). Measurements are in birds that underwent incubation in normoxic (N, white bars or circles) or hypoxic (H, black bars or circles) conditions. (**A**, n=12; **B–E**, n=25–32). *Significant effect of hypoxia (*P*<0.05). Student *t* test for unpaired data for **A–C**, and 2-way mixed-model ANOVA for **D** and **E**.

Compared with normoxic controls, hypoxic embryos showed significantly reduced survival rates, particularly between days 1 to 13 of incubation and from day 19 until hatching (percentage survival days 1–13: 95% (N) versus 54%* (H); days 13–19: 97% (N) versus 87%* (H); days 19–21: 86% (N) versus 23%* (H); *P<0.05; χ^2^ test). Compared with normoxic controls, hypoxic embryos showed elevated hematocrit and blood lactate levels but reduced blood glucose concentrations (Figure 1A; Table S1 in the Data Supplement). At hatching, hypoxic chicks were growth restricted, with significant reduction in body weight, crown rump length, BPD, and hindlimb lengths (Figure 1B; Table S1). This could not be explained by differences in egg size as there was no significant difference in egg weights at the start of incubation (N, 60.4 g ±1.4; H, 59.2 g ±1.4). A significant increase in BPD/body weight ratio (Figure 1C) suggests that the hypoxic hatchlings were asymmetrically growth restricted, with the reduction in head growth spared relative to the rest of the body. As the chickens grew to adulthood, chickens that had been incubated under hypoxia remained smaller, with persisting lower values for body weight and body mass index when compared with controls (Figure 1D and 1E; Table S1). However, by adulthood, the BPD/body weight ratio and brain-to-body-weight ratio had normalized, and tibial length was significantly increased in birds that had been hypoxic during incubation (Table S1).

### Arterial Blood Pressure and Vascular Function at Adulthood

**Figure 2.**
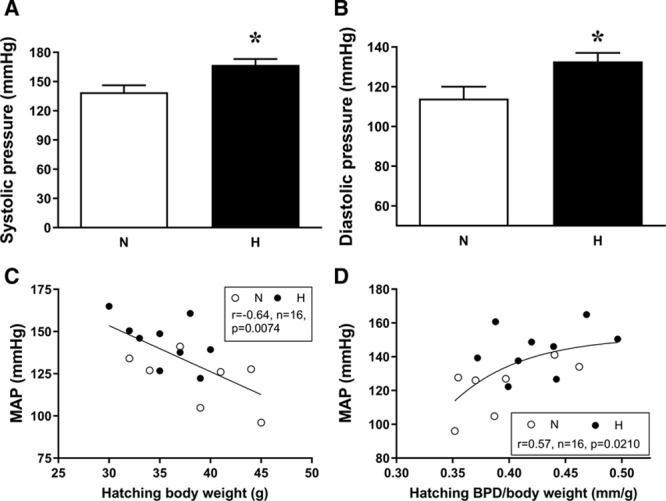
Arterial blood pressure in adult chickens. Values are mean±SEM for systolic blood pressure (**A**) and diastolic blood pressure (**B**) measured in vivo through indwelling arterial catheters placed in the descending aorta. **C**, Correlation between hatching body weight and mean arterial blood pressure (MAP) at adulthood. **D**, Correlation between hatchling biparietal diameter (BPD) relative to body weight and MAP at adulthood. Measurements are in adult chickens that underwent incubation in normoxic (N, white bars or circles) or hypoxic (H, black bars or circles) conditions. *Significant effect of hypoxia (*P*<0.05). Student *t* test for unpaired data (**A** and **B**, n=7–9) or 2-tailed Pearson correlation coefficient (**C** and **D**, n=16).

**Figure 3.**
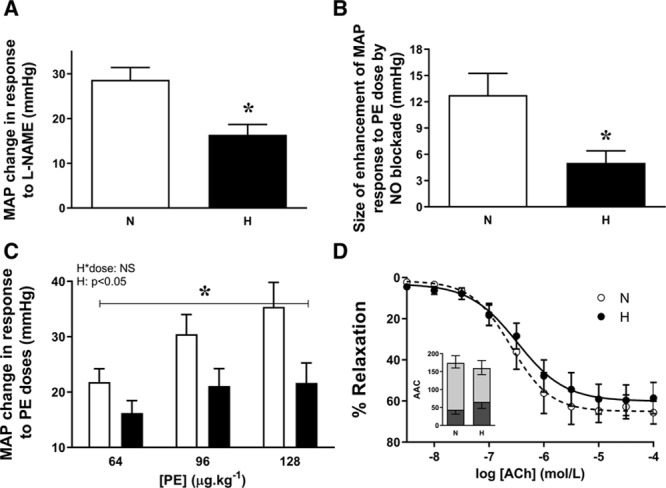
NO bioavailability, pressor responses, and vasomotor reactivity in adult chickens. Values are mean±SEM for in vivo blood pressure change in response to a dose of NG-nitro-L-arginine methyl ester (L-NAME; 100 mg·kg^−1^; **A**), the change in the size of the in vivo mean arterial blood pressure (MAP) response to a dose of phenylephrine (PE; 64 µg·kg^−1^) on the NO clamp compared with off the NO clamp (**B**), an in vivo dose pressor response curve for the change in MAP in response to 3 intravenous doses of PE (**C**), and the in vitro femoral vascular responsiveness to increasing doses of acetylcholine. The inset shows the area above the curve (AAC) contributions of NO-dependent relaxation (dark gray, downward error bars), NO-independent relaxation (light gray, downward error bars), and total AAC (dark and light gray bars combined, upward error bars; **D**). Measurements were made in adult chickens that underwent incubation in normoxic (N, white bars or circles) or hypoxic (H, black bars or circles) conditions. n=6 to 9 (**A** and **C**), n=5 to 6 (**B**), and n=7 to 11 (**D**). *Significant effect of hypoxia (*P*<0.05). Student *t* test for unpaired data for **A** and **B**, and 2-way mixed-model ANOVA for **C**. ACh indicates acetylcholine.

By adulthood, chickens that had been incubated under hypoxic conditions were hypertensive, with significant elevations in mean, systolic, and diastolic arterial pressure (Figure 2A and 2B; Table S2). The rate-pressure product was also significantly elevated, but there were no significant differences in basal heart rate or in femoral arterial blood flow, vascular resistance, or conductance (Table S2). Basal arterial blood gas measurements, hematocrit, and calculations of oxygen and glucose hindlimb delivery (content×blood flow) were also similar between the 2 groups (Table S1). There was a significant negative correlation between body weight at hatching and arterial blood pressure at adulthood (Figure 2C) and a significant positive correlation between head diameter relative to body weight ratio at hatching and arterial blood pressure at adulthood (Figure 2D). Administration of a bolus dose of the eNOS inhibitor L-NAME caused a rise in arterial blood pressure in all chickens. However, the size of the increment was significantly reduced in chickens that had been incubated in hypoxia (Figure 3A). Preventing basal de novo synthesis of NO with the NO clamp also increased the size of the MAP response to a dose of PE in all chickens, due to removal of opposing effects of NO. However, the size of the increment of this response was again significantly smaller in chickens that were incubated under hypoxic conditions (Figure 3B). Off the NO clamp, chickens that had been incubated in hypoxia showed a significantly reduced MAP response to PE doses (Figure 3C). Experiments ex vivo assessing vasomotor reactivity in isolated femoral arterial segments using in vitro wire myography showed no significant difference in the constrictor responsiveness to K+ or PE or in the dilator response to SNP or acetylcholine between the groups (Figure 3D; Table S2). There was also no significant difference in the contributions of NO-dependent and NO-independent mechanisms to acetylcholine-mediated femoral arterial vasorelaxation.

### Cardiac Structure and Function at Adulthood

**Figure 4.**
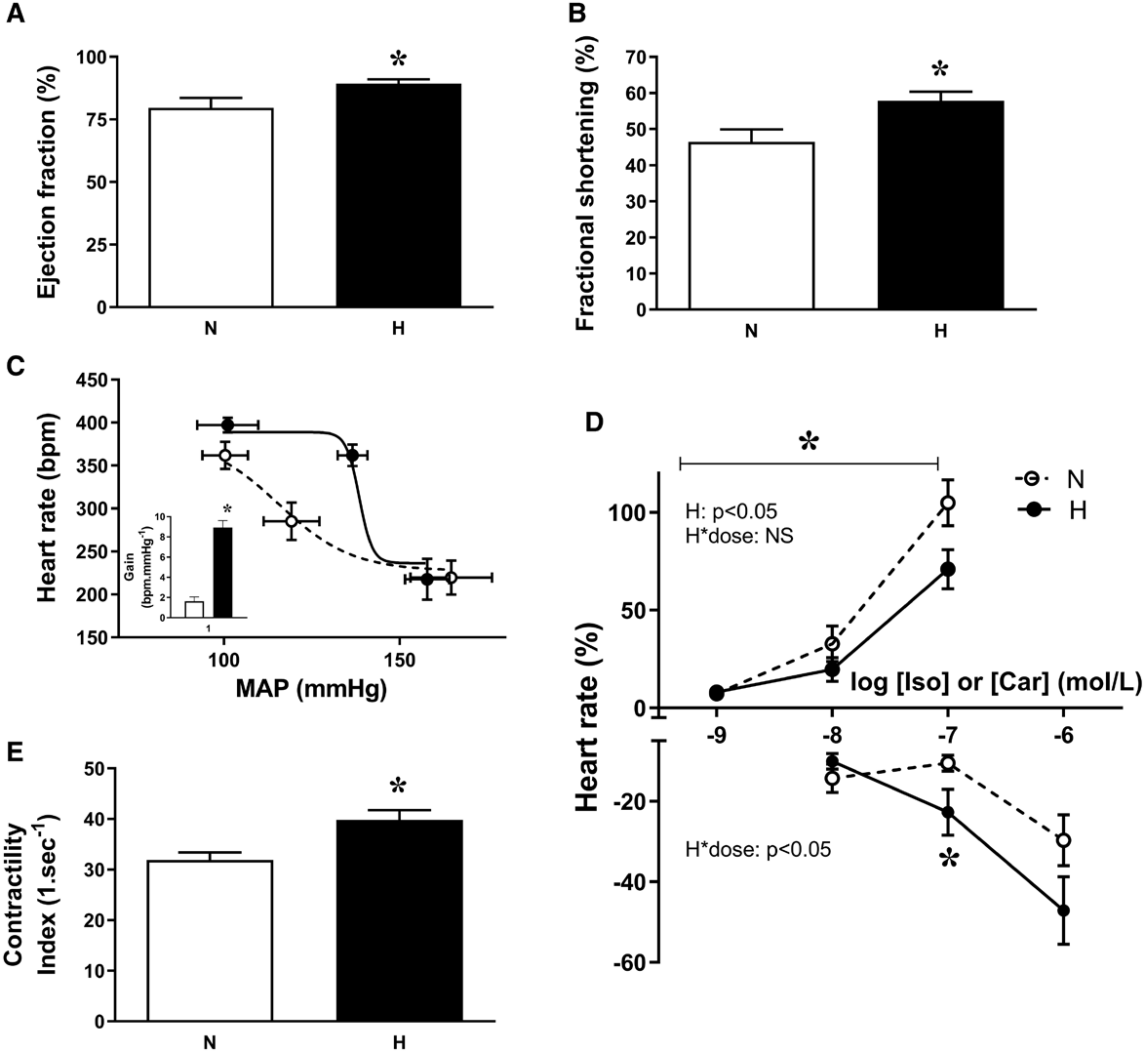
Cardiac function in adult chickens. Values are mean±SEM for ejection fraction (**A**), mean±SEM for fractional shortening (**B**), mean±x and y SEM for cardiac baroreflex function curves, with the inset showing the calculated gain of the response (**C**), mean±SEM for ex vivo chronotropic responses to increasing doses of isoprenaline (Iso) and carbachol (Car; **D**), and mean±SEM for the ex vivo cardiac contractility index (**E**) in adult chickens that underwent incubation in normoxic (N, white bars or circles) or hypoxic (H, black bars or circles) conditions. n=17 to 18 (**A** and **B**), n=7 to 9 (**C**), n=11 to 14 (**D**), and n=12 to 13 (**E**). *Significant effect of hypoxia (*P*<0.05). Student *t* test for unpaired data for **A–C** (inset) and **E**, and 2-way mixed-model ANOVA for **D**. For clarity only 3 points are shown in the cardiac baroreflex function curves. However, all points were taken into account for the fitting of the line and for the calculation of the cardiac baroreflex gain (Methods). MAP indicates mean arterial blood pressure.

At adulthood, echocardiography showed that hearts of adult chickens incubated under hypoxic conditions had significantly increased ejection fraction and fractional shortening (Figure 4A and 4B), as well as reduced LV lumenal diameter in systole (Table S2). Echocardiographic measurements of the right ventricle showed no significant differences (Table S2). A plot of cardiac baroreflex function measured in vivo revealed a significant increase in baroreflex gain in adult chickens incubated under hypoxic conditions (Figure 4C). In vitro, hearts of chickens that had been incubated in hypoxia showed blunted responsiveness to isoprenaline but enhanced responsiveness to carbachol (Figure 4D). The isolated heart also demonstrated increased ventricular contractility, with a significant increase in the contractility index and in dP/dt max (Figure 4E; Table S2).

**Figure 5.**
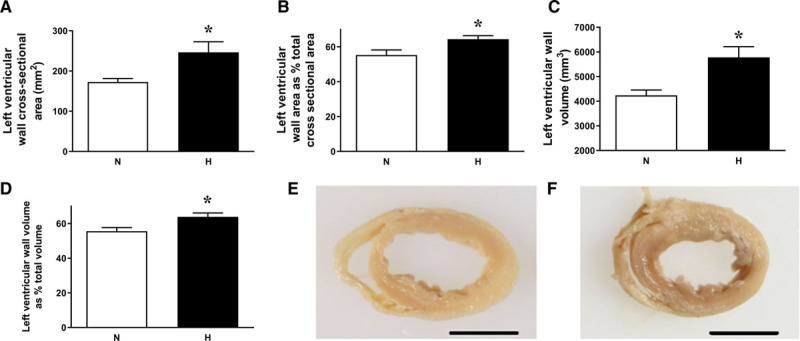
Cardiac morphology in adult chickens. Values are mean±SEM for left ventricular wall cross-sectional area (**A**), left ventricular wall cross-sectional area expressed as a percentage of total ventricular cross-sectional area (**B**), left ventricular wall volume (**C**), left ventricular wall volume expressed as a percentage of total ventricular volume (**D**), and representative images from a normoxic (**E**) and hypoxic (**F**) chicken. Scales bars represent 1 cm. Measurements are from adult chickens that underwent incubation in normoxic (N, white bars) or hypoxic (H, black bars) conditions. n=8 to 9 per group. *Significant effect of hypoxia (*P*<0.05). Student *t* test for unpaired data.

At adulthood, heart weight relative to body weight and LV weight/body weight ratio were both significantly greater in chickens that were incubated in hypoxic conditions (Table S1). The LV wall cross-sectional area and volume were significantly enhanced by hypoxic incubation, either when expressed in absolute terms or as a percentage of total cross-sectional area or total ventricular volume (Figure 5A through 5F). LV lumen areas and volumes were not significantly different between groups (Table S2). Right ventricular lumen area, only when expressed as a percentage of total cross-sectional area, was reduced in adults of hypoxic incubation, but there were no other differences in right ventricular histology (Table S2).

## Discussion

**Figure 6.**
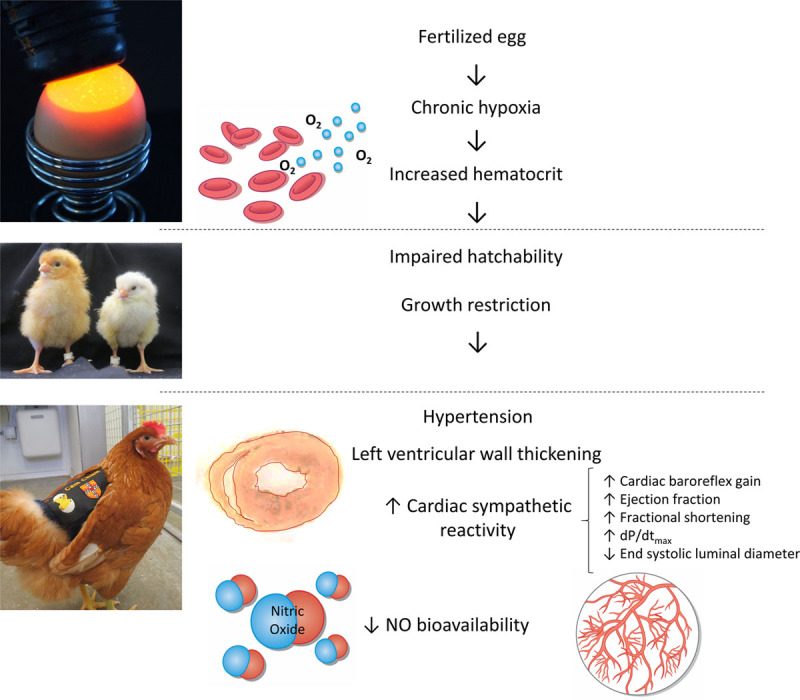
Summary figure. Hypoxic incubation increased hematocrit and induced asymmetrical growth restriction in the developing chick. By adulthood, chickens from hypoxic incubations remained smaller and were significantly hypertensive with left ventricular wall thickening, showing in vivo and ex vivo measures of increased cardiac sympathetic reactivity, represented by an increase in cardiac baroreflex gain, ejection fraction, fractional shortening, dP/dt_max_, and a decrease in left lumenal end systolic diameter. Additional mechanisms included reduced in vivo systemic NO bioavailability, represented by impaired in vivo pressor responses to NG-nitro-L-arginine methyl ester and by lower blood pressure reactivity to phenylephrine during the NO clamp in adult chickens, which were incubated under hypoxic incubations. Combined, the data support that developmental hypoxia alone can directly program hypertension in the adult offspring, independent of effects on the mother or placenta. Further, mechanisms underlying this phenotype include direct programming effects of developmental hypoxia on NO biology and sympathetic reactivity.

The data show that hypoxic incubation increased hematocrit and induced asymmetrical growth restriction in the developing chick. Hypoxia induces the activation of the HIF-1 (hypoxia-inducible factor-1), which binds to the hypoxia response elements of the erythropoietin gene, enhancing its transcription.^47^ Therefore, the data confirm that exposure of the chicken embryo to 13.5% to 14% oxygenation from the beginning of incubation induced chronic hypoxia. By adulthood, chickens from hypoxic incubations remained smaller and were significantly hypertensive with LV wall thickening, showing in vivo and ex vivo measures of increased cardiac sympathetic reactivity, represented by an increase in cardiac baroreflex gain, ejection fraction, fractional shortening, dP/dt_max_, and a decrease in left lumenal end systolic diameter. Additional mechanisms included evidence of reduced in vivo systemic NO bioavailability, represented by impaired in vivo pressor responses to L-NAME and by lower blood pressure reactivity to PE during the NO clamp in adult chickens that were incubated under hypoxic incubations. Therefore, combined, the data support the hypothesis tested that developmental hypoxia alone can directly program hypertension in the adult offspring, independent of effects on the mother or placenta. Further, mechanisms underlying this phenotype include direct programming effects of developmental hypoxia on NO biology and sympathetic reactivity (Figure 6).

When working with animal models of cardiovascular dysfunction programmed by adverse conditions before birth, the temporal profiles of cardiovascular development between species is a highly important translational consideration. Rats and mice are altricial species, in which cardiovascular maturation continues past birth, becoming completed by the second week of postnatal life.^35,48^ In contrast, chickens, nonhuman primates, and humans share similar temporal windows of precocial cardiovascular development and maturation.^35,49–51^ Therefore, in addition to permitting isolation of the direct developmental programming effects of chronic hypoxia on cardiovascular function at adulthood, cardiovascular data derived from avian models provide useful translation to the human situation.

According to the National Institute for Health and Care Excellence guidelines, human patients are diagnosed as hypertensive when the systolic/diastolic reading exceeds 140/90 mm Hg.^52^ There is no equivalent recommendation for chickens, and although direct comparisons should be made with caution, adult birds that had been incubated under hypoxic conditions in the present study had a mean increase in systolic and diastolic blood pressure values of 28.2 and of 18.8 mm Hg above control values, respectively. Data from human studies, such as the Framingham cohort, report that smaller increments in arterial blood pressure than those measured in the present study can significantly increase the risk of future cardiovascular dysfunction.^53^ Further, with each increment of systolic and diastolic pressure of 20 and 10 mm Hg, respectively, the human risk for an adverse cardiovascular event doubles and such patients require active preventative lifestyle modifications.^54^ Therefore, the magnitude of the hypertension in the present study would represent a significant risk of increased mortality from cardiovascular disease in humans and is especially striking given that these chickens are female and only young adults—2 factors that would tend to place them in a low-risk group for hypertensive diseases.^55,56^ The hypertension observed in this study contrasts with a previous study in which adult chickens that were incubated at high altitude were hypotensive, rather than hypertensive, at adulthood.^57^ However, in high-altitude hypoxia, the effect of hypobaric hypoxia is constant, continuing from in ovo development through to adulthood. Therefore, whether high altitude–induced effects on cardiovascular function in chickens at adulthood are due to hypoxia exposure in ovo or after hatching cannot be isolated. Several mammalian studies have also demonstrated that development under hypoxic conditions can program hypertension in the adult offspring.^16–18^ The present study highlights that a least a component of the hypertension programmed developmentally in mammals is due to the direct effects of hypoxia on the developing embryo, independent of any maternal or placental effects.

Interestingly, when biometry at hatching was related to arterial blood pressure at adulthood, data in the present study showed a significant negative relationship between body weight at hatching and MAP at adulthood, and a significant positive correlation between the BPD/body weight ratio at hatching and MAP at adulthood, with the values derived from hypoxic incubations extending toward the extremes of the relationships. These discoveries have many commonalities with the original findings of Barker et al^58^ who related reduced birth weight with increased rates of cardiovascular disease in human populations. Martyn et al^59^ reported that, in human babies, an increase in the ratio of the head circumference to birth weight correlates with an increased prevalence of stroke in adulthood. These correlations could also support that programmed hypertension in adulthood in the present model results from embryonic growth restriction rather than hypoxia in ovo. However, in the field of developmental programming, it is widely accepted that fetal growth restriction is a surrogate measure resulting from the adverse prenatal conditions that induce developmental programming, rather than a prerequisite for developmental programming. Support for this statement comes from several studies, including ours, that have reported programmed cardiovascular dysfunction by prenatal hypoxia independent of effects on fetal growth. Therefore, developmental hypoxia can programme cardiovascular dysfunction in the adult offspring with or without concomitant fetal growth restriction, depending on the time of onset, duration, and magnitude of the challenge. As an example, in rats, hypoxic pregnancy from days 15 to 21 of gestation (term is ≈21 days), reducing the O_2_ to 12%, promotes significant fetal growth restriction and programmes cardiovascular dysfunction in the adult offspring.^30^ Conversely, in rat pregnancy, hypoxic pregnancy from days 6 to 20 of gestation, reducing O_2_ to 13%, has no effect on fetal growth but still programmes cardiovascular dysfunction in the adult offspring.^20,60^

Data in the present study show that blocking basal NO production by administration of L-NAME caused a smaller increase in arterial blood pressure in chickens, which developed under hypoxic conditions. Further, under conditions of basal NO blockade with the NO clamp,^40–42^ treatment with the α_1_-adrenergic receptor agonist PE also led to a smaller increment in arterial blood pressure in chickens from hypoxic incubations. Combined, these in vivo data support that one mechanism underlying their hypertension is a reduced contribution of circulating NO. The presence of diastolic hypertension also corroborates an increase in peripheral vascular resistance and cardiac afterload in chickens from hypoxic incubations. In the current study, no significant difference was found in the dilator responsiveness of isolated femoral resistance vessels to SNP or to acetylcholine stimulation before and after L-NAME treatment. This may suggest that the impairment in NO biology is due to an alteration in NO bioavailability rather than vascular smooth muscle insensitivity to NO or to dysfunction in acetylcholine-dependent endothelial synthesis. This study could not determine any effects on the partial contributions of other endothelium-dependent vasodilators, such as prostacyclin or endothelium-derived hyperpolarizing factors because the vessels were preconstricted with potassium rather than with noradrenaline or PE. In a comprehensive study, Ruijtenbeek et al^61^ reported that in adult chickens, the relaxant responses to acetylcholine in isolated femoral arteries determined similarly via in vitro wire myography were highly variable and absent in almost 30% of the femoral arteries studied. However, in smaller side branches of the femoral artery, that study was able to show a reduced dilator response to acetylcholine after L-NAME treatment in adult chickens incubated in ovo. This supports reduced acetylcholine-induced NO release in isolated femoral arterial side branches in adult chickens that had been incubated under hypoxic conditions.^61^

Additional data in the present study show significant alterations in the autonomic regulation of cardiac function in birds from hypoxic incubations. The arterial blood pressure–heart rate relationship shows not only a rightward shift in the set point but a much steeper gradient of the relationship, suggesting cardiac sympathetic dominance—a finding confirmed by an increase in the calculation of the baroreflex gain. The echocardiography data showing an increase in ejection fraction and in fractional shortening with lower lumenal ventricular diameter at the end of systole coupled with the isolated Langendorff data reporting an increase in dP/dt_max_ and in the contractility index all further support cardiac sympathetic dominance, leading to systolic dysfunction. This suggests that hearts from adult birds raised from hypoxic incubations are working much harder to maintain cardiac output in the face of increased afterload—a response that may explain the increase in LV wall size. Increased sympathetic dominance to maintain cardiac function is unsustainable, and compensatory cardiac hypertrophy eventually leads to dilated cardiomypathy and eventual heart failure in humans.^62^ The switch from impaired α_1_-adrenergic receptor to enhanced muscarinic receptor responsiveness in hearts isolated from adult birds from hypoxic incubations and the impaired in vivo pressor response to α_1_-adrenergic receptor stimulation in the present study may, therefore, represent compensatory adaptive responses to buffer hypertension and ameliorate cardiovascular compromise. Previous studies in post-hatching chickens have reported dilated cardiomyopathy with reduced cardiac β-adrenoreceptor sensitivity as a result of hypoxic incubation.^63,64^ Two elegant studies by Ruijtenbeek et al^61,65^ reported sympathetic hyperinnervation of femoral arteries of both chick embryos and 15-week-old chickens that developed under chronic hypoxia. Differences between studies are likely the result of different age groups or different extents and durations of hypoxic exposure promoting varying phenotypic degrees of cardiovascular dysfunction. Analysis of cardiac and vascular adrenoreceptor expression and catecholamine content in the current model would be useful incremental knowledge. However, in the present study, this could not be done, as naive frozen tissues, which had not been manipulated by the in vivo or in vitro experiments, were not generated. Previous studies in mammals have demonstrated that exposure to chronic hypoxia during pregnancy can program changes in cardiac structure and function at adulthood, reporting an increase in sympathetic dominance.^20,66–68^ In humans, there is also evidence of cardiac dysfunction and structural changes in offspring of complicated pregnancy.^69^ Past^61,63–65^ and present studies in birds, therefore, highlight that a least a component of the cardiovascular dysfunction programmed developmentally by prenatal hypoxia in mammals is due to the direct effects of hypoxia on the embryo, independent of any maternal or placental effects. Interestingly, in the present study, there was little evidence of any effect of hypoxia in ovo on right ventricular structure or function at adulthood. Previous studies in mammals^68^ and chickens^70^ have suggested that development under hypoxic conditions can programme pulmonary hypertension in adulthood. It is possible that the level of hypoxic incubation in the present study was insufficient to program tissue remodeling associated with pulmonary hypertension. However, the data highlight that left heart dysfunction can be programmed independent of effects on right heart function.

Reduced birth or hatching weight is a well-documented consequence of hypoxic development in avian and mammalian animal models,^19,24,26,32,63,71,72^ as well as in human pregnancy compromised by high altitude or by placental insufficiency or preeclampsia.^73–76^ Clinical studies in humans have reported that a major factor promoting fetal growth restriction in pregnancy complicated by the chronic hypoxia of high altitude is altered glucose metabolism in the placenta, leading to fetal hypoglycemia.^77–79^ However, our avian model demonstrates both embryonic hypoglycemia and growth restriction independent of the existence of a placenta. This suggests that the mechanisms inducing fetal growth restriction in human highland pregnancy may not be entirely mediated by the placenta or by a reduction in fetal glucose delivery. The present data, therefore, underscore the potential for a direct influence of fetal oxygenation in the regulation of fetal growth.^71^

## Perspectives

In mammals, there are several routes for developmental programming of an increased cardiovascular risk in the adult offspring in adverse pregnancy. The maternal adaptation to pregnancy may be suboptimal. For instance, a maternal cardiogenic origin of preeclampsia has been proposed, whereby women with low prepregnancy cardiac output are at greater risk of placental malperfusion and increased placental vascular resistance, thereby promoting fetal growth restriction and maternal hypertension.^80,81^ In turn, offspring of human hypertensive pregnancies have increased cardiovascular risk factors during childhood.^82^ Alternatively, the placenta, at the interface between the mother and the fetus, may alter its metabolism affecting resource distribution, it may alter its capacities as a protective barrier, and it may modify the secretion of hormones and agents into the fetal and maternal circulation, which may trigger an increased risk of cardiovascular dysfunction in the offspring, as well as in the mother.^83–85^ For example, the release of cytotrophoblast-derived exosomes is increased under hypoxic conditions.^86^ Therefore, in mammals, the partial effects of suboptimal pregnancy on the mother, the placenta, and the fetus and their contributions to developmental programming are difficult to disentangle. By simplifying the layers of complexity in mammals and employing the chicken embryo model, this study provides a significant conceptual advance to the field, showing that isolated challenges to the developing embryo independent of effects on the mother and the placenta can by themselves program an increased risk of cardiovascular dysfunction in the adult offspring. Therefore, this study provides a better understanding of the mechanisms involved in the developmental programming of adult-onset hypertension, enabling improved isolation of suitable and rational interventional therapies.

## Acknowledgments

We are extremely grateful to the staff of the University of Cambridge Biological Services for helping with the maintenance of the chickens at the Barcroft Centre.

## Sources of Funding

K.L. Skeffington was a student on the University of Cambridge Clinical School Metabolic and Cardiovascular Disease PhD programme. The study was funded by a British Heart Foundation project grant (PG/10/99/28656 to D.A. Giussani). D.A. Giussani is the Professor of Cardiovascular Developmental Physiology and Medicine at the Department of Physiology, Development and Neuroscience at the University of Cambridge; Professorial Fellow and Director of Studies in Medicine at Gonville and Caius College; a Lister Institute Fellow; and a Royal Society Wolfson Research Merit Award holder.

## Disclosures

None.

## Supplementary Material



## Novelty and Significance

### What Is New?

- Fetal development under lower than normal oxygenation, chronic fetal hypoxia, programs a greater risk of hypertension in the adult offspring.
- However, whether this is due to effects of chronic hypoxia on the mother, placenta, or fetus is difficult to disentangle.
- We used the chicken embryo model system to isolate the direct effects of hypoxic development in promoting hypertension in the adult offspring.

### What Is Relevant?

- The animals in this study developed significant hypertension in adult life, which was not caused by any genetic, postnatal lifestyle factors or effects on the mother or placenta.
- Therefore, investigation of mechanisms underlying the programming of adult-onset hypertension by direct exposure of the developing embryo to adverse conditions before birth is important for the design of new treatments and preventative strategies against hypertension.

### Summary

We have isolated a direct effect of chronic fetal hypoxia in programming hypertension in the adult offspring via mechanisms altering NO signaling and sympathetic reactivity.
