# Supplementary figures and images for: Hypertension Programmed in Adult Hens by Isolated Effects of Developmental Hypoxia In Ovo
Source: Hypertension. 2020 Jun 15;76(2):533–44. doi: 10.1161/HYPERTENSIONAHA.120.15045 (PMC7340221; doi:10.1161/HYPERTENSIONAHA.120.15045)

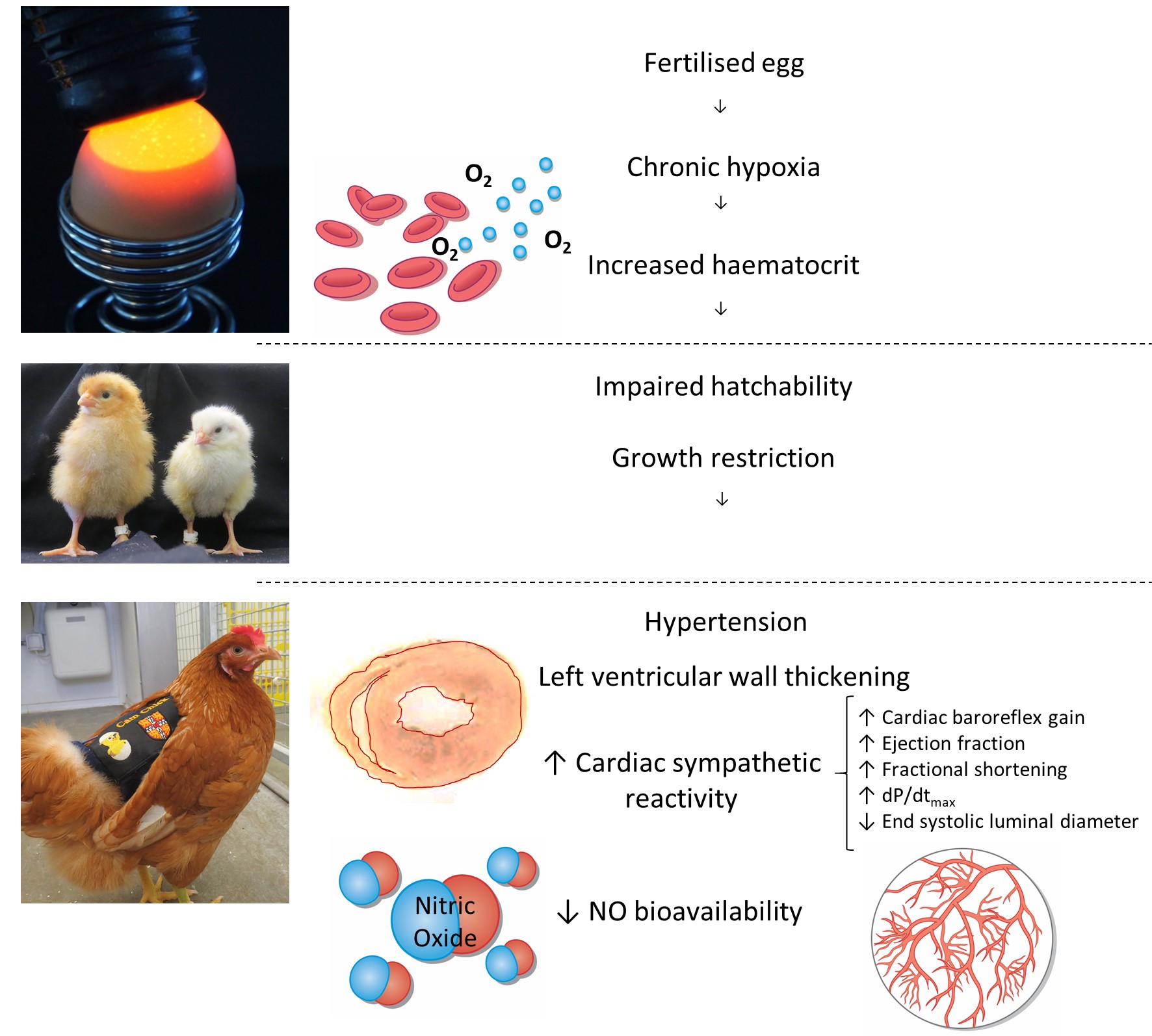

Supplement: Supplementary file 1 [file hyp-76-533-s001.jpg]
