# Supplementary material for: Hypertension Programmed in Adult Hens by Isolated Effects of Developmental Hypoxia In Ovo
Source: Hypertension. 2020 Jun 15;76(2):533–44. doi: 10.1161/HYPERTENSIONAHA.120.15045 (PMC7340221; doi:10.1161/HYPERTENSIONAHA.120.15045)
Supplement: Supplementary file 2 [file hyp-76-533-s002.pdf]

ONLINE SUPPLEMENT

HYPERTENSION PROGRAMMED IN ADULT HENS BY ISOLATED EFFECTS OF DEVELOPMENTAL HYPOXIA IN OVO

Katie L Skeffington[1], Christian Beck[1], Nozomi Itani[1], Youguo Niu[1], Caroline J Shaw[1,2] and Dino A Giussani[1]

[1]Department of Physiology, Development & Neuroscience, University of Cambridge, Downing Street, Cambridge, CB2 3EG, UK

[2] Institute of Reproductive and Developmental Biology, Imperial College London, London, UK

Corresponding author: Prof. Dino A Giussani, PhD
Department of Physiology, Development & Neuroscience
University of Cambridge
CB2 3EG
Tel: +44 1223 333894
Fax: +44 1223 333840
E-mail: dag26@cam.ac.uk

	Measurement	Normoxic	Hypoxic
**Blood chemistry of chicks at day 19 of incubation** *(n=12-19)*	**Lactate (mmol.L^{-1})**	0.89 ± 0.08	1.35 ± 0.14 *
	**Glucose (mmol.L^{-1})**	7.55 ± 0.19	6.71 ± 0.33 *
**Biometry of chicks at hatching** *(n=25-31)*	**CRL (cm)**	9.1 ± 0.1	8.8 ± 0.1 *
	**BPD (mm)**	15.5 ± 0.1	15.1 ± 0.1 *
	**Femur length (mm)**	25.2 ± 0.3	24.5 ± 0.4
	**Tibia length (mm)**	33.2 ± 0.3	31.9 ± 0.4 *
	**Metatarsal length (mm)**	25.7 ± 0.2	24.1 ± 0.2 *
**Biometry of adult chickens** *(n=7-20)*	**Body weight (g)**	1.59 ± 0.03	1.47 ± 0.04 *
	**CRL (cm)**	37.57 ± 0.48	37.79 ± 0.52
	**BPD (mm)**	2.67 ± 0.04	2.75 ± 0.0.6
	**BPD/body weight (mm.g^{-1})**	1.71 ± 0.05	1.91 ± 0.09
	**Femur length (mm)**	10.24 ± 0.22	10.39 ± 0.13
	**Tibia length (mm)**	12.95 ± 0.19	13.80 ± 0.11 *
	**Metatarsal length (mm)**	9.48 ± 0.12	9.51 ± 0.06
	**Heart weight (g)**	12.33 ± 0.63	12.19 ± 0.58
	**Heart weight/body weight (g.kg^{-1})**	7.52 ± 0.92	8.64 ± 1.43 *
	**Left ventricular weight (g)**	6.40 ± 1.69	6.60 ± 0.63
	**Left ventricular weight/body weight (g.kg^{-1})**	3.80 ± 0.22	4.54 ± 0.18 *
	**Right ventricular weight (g)**	2.59 ± 0.73	2.53 ± 0.55
	**Right ventricular weight/body weight (g.kg^{-1})Brain weight (g)**	1.68 ± 0.17	1.74 ± 0.14
	**Brain weight/body weight (g.kg^{-1})**	2.94 ± 0.06	2.89 ± 0.06
	**Liver weight (g)**	1.84 ± 0.04	1.97 ± 0.09
	**Liver weight/body weight (g.kg^{-1})**	37.04 ± 1.53	34.90 ± 1.42
		23.79 ± 0.82	24.50 ± 1.10
**Blood chemistry of adult chicken** ***(n=6-9)***	**pH**	7.53 ± 0.02	7.52 ± 0.01
	**$PaCO_2$ (mmHg)**	30.1 ± 1.3	30.8 ± 1.5
	**PaO_2 (mmHg)**	118.0 ± 2.6	118.9 ± 3.8
	**Hb (g.dL^{-1})**	6.2 ± 0.3	6.6 ± 0.2
	**Hematocrit (%)**	19.4 ± 0.3	19.9 ± 0.4
	**HbO_2 (%)**	94.0 ± 1.3	93.6 ± 0.4
	**Lactate (mmol.L^{-1})**	0.98 ± 1.14	1.08 ± 0.12
	**Glucose (mmol.L^{-1})**	12.4 ± 0.8	11.3 ± 0.6
	**Femoral oxygen delivery (µmol.min^{-1})** ***(n=5)***	90.0 ± 14.0	123.1 ± 12.2
	**Femoral glucose delivery (µmol.min^{-1})** ***(n=5-7)***	349.1 ± 47.2	342.3 ± 53.3

Supplementary Table S1. Biometry and blood chemistry of hatchlings and adult chickens. Values are mean ± SEM from hatchlings and adult chickens which underwent incubation in

normoxic or hypoxic conditions. * represents a significant effect of hypoxia ($p<0.05$). Student's *t*-test for unpaired data.

	Measurement	Normoxic	Hypoxic
In vivo basal cardiovascular function *(n=7-9)*	Mean arterial blood pressure (mmHg)	122 ± 6	145 ± 5*
	Heart rate (beats.min^{-1})	266 ± 19	288 ± 14
	Pulse pressure (mmHg)	25.1 ± 4.2	34.6 ± 4.0
	Mean femoral flow (ml.min^{-1}) (n=5-7)	25.0 ± 3.7	28.5 ± 3.5
	Rate pressure product ((beats.min^{-1}).mmHg)	37033 ± 3062	47638 ± 2243*
	Femoral arterial resistance (mmHg.(ml.min^{-1})$^{-1}$) (n=5-7)	5.26 ± 0.79	5.52 ± 0.82
	Femoral arterial conductance ((ml.min^{-1}).mmHg^{-1}) (n=5-7)	0.21 ± 0.04	0.20 ± 0.03
Ex vivo wire myography *(n=8-10)*	Internal circumference	1830 ± 161	1625 ± 120
	K^+ AAC (normalised to internal circumference)	0.37 ± 0.05	0.41 ± 0.08
	Phenylephrine curve AAC	147.3 ± 18.7	160.9 ± 36.1
	Sodium nitroprusside curve AAC	121.3 ± 5.1	114.6 ± 17.5
In vivo echocardiography *(n=11-18)*	Cardiac output (ml.min^{-1})	702.7 ± 65.0	765.0 ± 58.2
	Stroke volume (ml)	1.93 ± 0.17	2.24 ± 0.19
	Left ventricular		
	Posterior wall diameter systole (mm)	5.40 ± 0.37	4.57 ± 0.26
	Posterior wall diameter diastole (mm)	4.04 ± 0.20	3.48 ± 0.24
	Lumen diameter systole (mm)	6.12 ± 0.63	4.50 ± 0.36 *
	Lumen diameter diastole (mm)	10.96 ± 0.48	10.73 ± 0.40
	End systolic volume (ml)	0.54 ± 0.15	0.29 ± 0.06
	End diastolic volume (ml)	2.84 ± 0.32	2.62 ± 0.25
	Intraventricular septum		
	Wall diameter systole (mm)	4.56 ± 0.33	4.11 ± 0.33
	Wall diameter diastole (mm)	2.99 ± 0.22	2.56 ± 0.18
	Mitral valve		
	E (m.sec^{-1})	0.89 ± 0.07	0.78 ± 0.05
	A (m.sec^{-1})	0.40 ± 0.03	0.39 ± 0.04
	Pressure gradient (mmHg)	3.38 ± 0.48	2.57 ± 0.36
	Right ventricular		
	Free wall diameter systole (mm)	2.47 ± 0.24	2.26 ± 0.28
	Free wall diameter diastole (mm)	1.54 ± 0.13	1.67 ± 0.15
	Lumen diameter systole (mm)	1.75 ± 0.18	2.12 ± 0.29
	Lumen diameter diastole (mm)	4.65 ± 0.75	4.15 ± 0.53
	Pulmonary trunk lumenal diameter (mm)	4.65 ± 0.36	3.96 ± 0.27
	Tricuspid valve		
	E (m.sec^{-1})	0.83 ± 0.05	0.92 ± 0.07
	A (m.sec^{-1})	0.42 ± 0.03	0.42 ± 0.04
	Pressure gradient (mmHg)	2.87 ± 0.34	3.21 ± 0.50
	Regurgitant jet (mmHg)	0.92 ± 0.15	1.18 ± 0.25
	Pulmonary valve		
	Pressure gradient (mmHg)	4.86 ± 0.37	4.61 ± 0.53

	Maximal velocity ($m.sec^{-1}$)	1.13 ± 0.07	1.06 ± 0.06
Ex vivo	Coronary flow rate ($ml.min^{-1}$)	38.3 ± 2.8	41.3 ± 2.9
Langendorff preparation	Coronary flow rate/heart weight ($ml.min^{-1}. g^{-1}$)	3.12 ± 0.25	3.36 ± 0.31
(n=11-14)	Heart rate ($beats.min^{-1}$)	155.6 ± 8.5	171.7 ± 10.7
	Cycle duration (sec)	0.397 ± 0.027	0.358 ± 0.024
	Systolic duration (sec)	0.148 ± 0.005	0.138 ± 0.007
	Diastolic duration (sec)	0.249 ± 0.024	0.220 ± 0.018
	Left ventricular		
	Developed pressure (mmHg)	93.3 ± 7.6	99.5 ± 3.3
	Rate pressure product (($beats.min^{-1}$) .mmHg))	16247 ± 1720	18730 ± 909
	Tension time index ($mmHg.sec^{-1}$)	10.0 ± 0.9	9.8 ± 0.8
	End diastolic pressure (mmHg)	9.96 ± 1.51	7.67 ± 1.17
	Tau (sec)	0.032 ± 0.002	0.029 ± 0.002
	dP/d*t* min ($mmHg.sec^{-1}$)	-1582 ± 130	-1835 ± 91
	dP/*d*t max ($mmHg.sec^{-1}$)	1796 ± 183	2311 ± 107 *
	IRP dP/d*t* min ($mmHg.sec^{-1}$)	-1087 ± 91	-1250 ± 58
Histology	Left Ventricular		
(n=7-9)	Wall width (mm)	4.6 ± 0.4	5.3 ± 0.6
	Lumen cross-sectional area (mm^2)	66.7 ± 15.9	57.5 ± 13.5
	Lumen cross-sectional area as % total cross sectional area	18.4 ± 3.9	15.4 ± 3.9
	Lumen volume (mm^3)	1423 ±.351	1262 ± 315
	Lumen volume (% total ventricular volume)	15.1 ± 3.1	14.2 ± 3.6
	Right ventricular		
	Wall width (mm)	1.5 ± 0.1	1.7 ± 0.2
	Wall cross-sectional area (mm^2)	52.2 ± 8.1	52.5 ± 10.8
	Lumen cross-sectional area (mm^2)	32.5 ± 7.0	27.5 ± 9.4
	Wall cross-sectional area as % total cross sectional area	15.1 ± 1.7	14.0 ± 2.7
	Lumen cross-sectional area as % total cross sectional area	11.2 ± 2.1	4.8 ± 1.0*
	Wall volume (cm^3)	1.23 ± 0.16	1.31 ± 0.83
	Lumen volume (cm^3)	1.06 ± 0.24	0.72 ± 0.25
	Wall (% total ventricular volume)	15.0 ± 1.3	14.3 ± 1.8
	Lumen (% total ventricular volume)	11.9 ± 1.9	7.6 ± 2.0

Supplementary Table S2. Cardiovascular structure and function in adult chickens. Values are mean ± SEM for measurements obtained by *in vivo* studies, echocardiography, the Langendorff preparation, wire myography and histological analysis in adult chickens which underwent incubation in normoxic or hypoxic conditions. * represents a significant effect of hypoxia ($p<0.05$). Student's *t*-test for unpaired data.
