# Supplementary material for: Hypertension Programmed in Adult Hens by Isolated Effects of Developmental Hypoxia In Ovo
Source: Hypertension. 2020 Jun 15;76(2):533–44. doi: 10.1161/HYPERTENSIONAHA.120.15045 (PMC7340221; doi:10.1161/HYPERTENSIONAHA.120.15045)
Supplement: Supplementary file 3 [file hyp-76-533-s003.pdf]

*** Short In Vivo Checklist**

AHA - Preclinical Animal Testing: Prevention of bias is important for experimental cardiovascular research. ***This short checklist must be completed, and the answers should be clearly presented in the manuscript as well.*** *The checklist will be used by reviewers and editors but will not be published. If a revision is requested, you will be required to complete at revision submission a more detailed checklist that will be published with the accepted article.*

This study involves animals:
Yes

Animals

Species, age, sex, strains, and sources of animals are described: Yes

Randomization

Randomization and allocation concealment were performed: Yes

Blinding

Blinding was performed: Yes

Inclusions and Exclusions (a)

Specific criteria for inclusions and exclusions are specified: Yes

Inclusions and Exclusions (b)

Criteria for inclusions and exclusions were set before the study: Yes

Reporting of Excluded Animals

All animals excluded after the randomization are reported: Yes

Statistical Methods

Statistical Methods are described: Yes

Date completed: 05/01/2020 06:18:47
User pid: 7421
